# Removal of Pharmaceutical Products in a Constructed Wetland

**DOI:** 10.15171/ijb.1223

**Published:** 2016-12

**Authors:** Nihan Özengin, Ayse Elmaci

**Affiliations:** Department of Environmental Engineering, Gorukle Campus, Faculty of Engineering, Uludag University, 16059, Bursa, Turkey

**Keywords:** Carbamazepine, Constructed Wetland, Ibuprofen Leca, *Phragmites australis*, Sulfadiazine

## Abstract

**Background:**

There is growing interest in the natural and constructed wetlands for wastewater treatment. While nutrient removal in wetlands has been extensively investigated, information regarding the degradation of the pharmaceuticals and personal care products (PPCPs) has only recently been emerging. PPCPs are widely distributed in urban wastewaters and can be removed to some extent by the constructed wetlands. The medium-term (3-5 years) behavior of these systems regarding PPCP removal is still unknown.

**Objectives:**

The efficiency of a Leca-based laboratory-scale constructed wetland planted with *Phragmites australis* (Cav.) Trin. Ex. Steudel in treating an aqueous solution of the pharmaceuticals, namely, carbamazepine, ibuprofen, and sulfadiazine, was to investigate.

**Materials and Methods:**

The two pilot-scale constructed wetlands (CW) were operated in parallel; one as an experimental unit (a planted reactor with *P. australis*) and the other as a control (an unplanted reactor with Leca). Pretreatment and analyses of the carbamazepine, ibuprofen, sulfadiazine, and tissue samples (Leca, *P. australis* body and *P.australis* leaf) were conducted using HPLC.

**Results:**

The carbamazepine, ibuprofen, and sulfadiazine removal efficiencies for the planted and unplanted reactors were 89.23% and 95.94%, 89.50% and 94.73%, and 67.20% and 93.68%, respectively. The Leca bed permitted an efficient removal. Leca has a high sorption capacity for these pharmaceuticals, with removal efficiencies of 93.68-95.94% in the unplanted reactors.

**Conclusions:**

Sorption processes might be of a major importance in achieving efficient treatment of wastewater, particularly in the removal of organic material that are resistant to biodegradation, in which case the materials composing the support matrix may play an important role. The results obtained in the present study indicate that a constructed wetland with Leca as a substrate and planted with *P. australis* is effective in the treatment of wastewater contaminated with carbamazepine, ibuprofen, and sulfadiazine.

## 1. Background

As emerging contaminants, pharmaceuticals and personal care products (PPCPs) are of increasing environmental concern because of their widespread release into the aquatic environments, their persistence, and increasing evidence of their ecotoxicological effects (1-4). Variable concentrations of these compounds have been detected in the surface, ground, and coastal waters into which treated sewage effluents are drained (5-7). Predictive models that estimate PPCPs persistence as a function of the compound’s physical and chemical properties have been developed with varying degrees of success for specific water treatment technologies (8-10). Unfortunately, up to now not a single approach has been emerged that could accurately predict PPCP removal during wastewater treatment over a wide range of treatment technologies, water quality condition, and PPCP compounds as well as classes. One of the most highly researched PPCPs is ibuprofen, an over-thecounter anti-inflammatory drug. Sixty-five reports have been presented by the conventional treatment facilities for removing the influent and effluent concentrations of the ibuprofen reporting roughly 70% efficiencies greater than 1^-log10^ for removal of the treated wastewaters (2).

Carbamazepine at a concentration of μg.L^-1^ levels has been measured by many researchers. Working on surface water samples collected in Berlin, concentrations of the carbamazepine at an amount of 1.075 μg.L^-1^ was measured (11). The highest reported concentration of carbamazepine in the municipal wastewater was 56 μg.L^-1^ by Mersmann *et al*. (12).

Sulfadiazine belongs to one of the largest classes of the antibiotics applied in Europe in the animal husbandry. It appears to be quickly transported to the surface water, whereas the transport to the highly sorptive substances appears to be much slower, with concentrations measured in drainage outfalls many months after application (13).

Constructed wetlands are land-based wastewater treatment systems which are consisted of the shallow ponds, beds, or trenches that contain floating or emergentrooted wetland vegetation (14). In subsurface-horizontalflow constructed wetlands (SSFs), wastewater infiltrates through vegetated gravel beds confined by a liner. The wastewater treatment relies on biological, chemical, and physical processes occurring in a natural environment. The potential of SSFs to remove contaminants from urban wastewater has attracted increasing interest over the past decade, particularly for treating wastewater from small communities (15-20).

*Phragmites australis* (Cav.) Trin. Ex. Steudel, also known as the common reed, is a perennial wetland grass that can grow to 15 feet in height. The invasive variety of phragmites creates tall, dense stands that degrade wetlands and coastal areas by crowding out native plants and animals, blocking shoreline views, and reducing access for swimming, fishing, and hunting. The dry plant material of this variety can also be a fire hazard. It spreads rapidly because of its vigorous rhizomes (horizontal roots that produce new shoots) and can grow more than six feet per year (21,22).

Optimization of a constructed wetland for removal of particular pollutants could be achieved by careful selection of its components, such as the plant species used, and the materials that compose the support matrix. A suitable choice of the latter is especially important for the removal of non-biodegradable compounds (including phenolic compounds, pharmaceuticals, and pesticides) from wastewater, for which sorption processes can play a major role (23-26). In the previous studies, lightweight expanded clay aggregate (Leca) materials have been shown to be suitable for the development of the plants and microorganisms in the constructed wetlands, as well as exhibiting a high capacity for adsorbing many types of the organic molecules (27-33).

Limited information exists about the removal of pharmaceuticals from wastewater in engineered natural systems such as constructed wetlands. The studies conducted to date have pertained primarily to herbicides, pesticides (34-36), and surfactants (37,38). Only a few studies have focused on pharmaceutical removal from wastewater in constructed wetlands (18,39,40).

## 2. Objectives

The study described herein is one of only a few that have investigated the removal of pharmaceuticals from a synthetic domestic wastewater using a laboratoryscale pilot subsurface-flow constructed wetland. In this study, the efficiency of a Leca-based laboratoryscale constructed wetland planted with *P. australis* treating an aqueous solution of pharmaceuticals, namely, carbamazepine, ibuprofen, and sulfadiazine, was evaluated. These pharmaceuticals were chosen on the basis of their high production volume and widespread use. Carbamazepine is the most commonly used drug treatment for epilepsy worldwide. Ibuprofen is the main component of drugs for pain relief, fever, and rheumatic symptoms. Sulfadiazine is an antibacterial drug that is widely used in the veterinary medicine, especially for poultry and fish. In this study, an assessment was carried out on the suitability and performance of a Leca-based laboratory-scale constructed wetland planted with *P. australis* treatment of the wastewater contaminated with these three pharmaceuticals.

## 3. Materials and Methods

### 3.1. Reagents and Materials

Carbamazepine, ibuprofen, and sulfadiazine were purchased from Sigma-Aldrich (Steinheim, Germany). Gravel (grain sizes of 10-15 mm) and Leca (granulometric grades 2/4 and 3/8), which were used for the support matrix of the constructed wetland, were supplied by a landscaping firm in Turkey. Prior to use, the gravel and Leca were washed several times with water to remove fine particles and suspended solids.

### 3.2. Experimental Set-up and Operating Conditions of Constructed Wetland

The constructed wetland units were operated in a subsurface-flow mode, in which all influent wastewater was forced to flow through the constructed wetland beds, and no wastewater flowed above the constructed wetland beds. Two pilot-scale subsurface-flow constructed wetland units were constructed from stainless steel, each with dimensions of 0.2×2.45×0.45 m (width × length × depth). A layer of Leca 27 cm thick was used as the support medium in these units. To facilitate distribution, gravel was used in the influent and effluent zones. These two pilot-scale constructed wetlands were operated in parallel, one as an experimental unit (a planted reactor with *P. australis*) and the other as a control (an unplanted reactor with Leca). *P. australis* was planted at a density of 4 m^-2^. Wastewater was fed continuously to the constructed wetlands units to acclimatize the soil microbes and support growth of the cattail plants. All physical, chemical, and biological parameters of the wastewater were analyzed according to the standard methods (41).

### 3.3. Support Media (Lightweight Expanded Clay Aggregate, Leca)

Natural lightweight aggregates are industrial raw materials, usually formed as the product of volcanic porous and large mass distributions. Industrially produced synthetic aggregates include a wide variety of products, generally known by their trade names. The sintering process occurs rapidly.

The specific volume increases at the temperatures between 1100ºC and 1300ºC for clay and shale, which are generally called swell able clays. Raw materials for widely used expanded clays include early sintered clay, sandy clay, and shale. The outer surfaces of sintered porous ceramic products are slightly hard and have a pyroclastic structure that forms a shell. This lightweight aggregate material formation is used in the construction industry in lightweight structural elements (42).

### 3.4. Operation of the System and Sample Collection

All systems were operated in a continuous mode. The hydraulic retention time (HRT) of the system was 3 days. This experimental stage was carried out when the reeds were quite well developed. Twenty liters of the synthetic wastewater were spiked daily with each pharmaceutical compound to obtain preselected concentrations of the carbamazepine, ibuprofen, and sulfadiazine. Because there hasn’t been a study related to the monitoring pharmaceuticals in the inland waters in Turkey, the influent concentrations of the pharmaceutical compound were set on the basis of the monitoring studies reported in the literature. At the end of the HRT, influent and effluent composite samples were collected. In the planted reactor, the longer-term monitoring was conducted than in the control reactor. Samples were collected in one-liter amber glass bottles and kept refrigerated during transport to the laboratory, where they were stored at 4ºC until analysis.

### 3.5. Analysis of Pharmaceuticals and Tissues

Pretreatment and analyses of the carbamazepine, ibuprofen, sulfadiazine, and tissue samples (Leca, *P. australis* body and *P. australis* leaf) were conducted in an accredited laboratory in Spain (Ecosur Laboratories) using a dual-pump HPLC equipped with a UV diode array detector.

## 4. Results

### 4.1. Carbamazepine Assays

**Figure 1:**
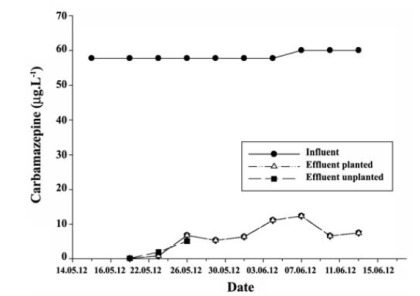
Concentrations of carbamazepine in the influent and effluent of the planted and unplanted reactors

The average carbamazepine concentration in the subsurface-flow wetland system was 58.43 μg.L^-1^ in the influent, 6.29 μg.L^-1^ in the planted reactor, and 2.37 μg.L^-1^ in the unplanted reactor (Figure 1). The carbamazepine removal efficiencies for the planted and the unplanted reactors were 89.23% and 95.94%, respectively.

### 4.2. Ibuprofen Assays

**Figure 2:**
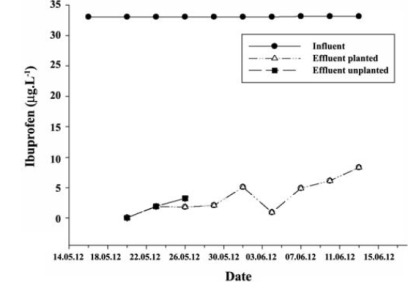
Concentrations of ibuprofen in the influent and effluent of the planted and unplanted reactors

Figure 2 shows the ibuprofen concentrations in the influent and effluent of the planted and the unplanted reactors. The average ibuprofen concentrations in the subsurface-flow wetland system were 33.07 μg.L^-1^ in the influent, 3.47 μg.L^-1^ in the planted reactor, and 1.74 μg.L^-1^ in the unplanted reactor. The ibuprofen removal efficiencies for the planted and unplanted reactors were 89.50% and 94.73%, respectively.

### 4.3. Sulfadiazine Assays

**Figure 3:**
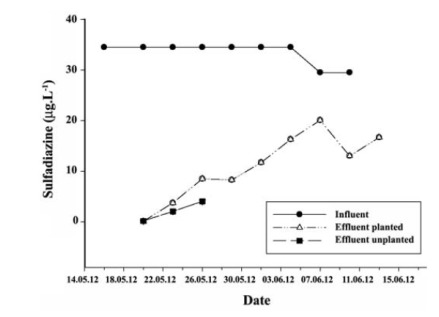
Concentrations of sulfadiazine in the influent and effluent of the planted and unplanted reactors

The average sulfadiazine concentrations in the subsurface-flow wetland system were 33.39 μg.L^-1^ in the influent, 10.95 μg.L^-1^ in the planted reactor, and 2.11 μg.L^-1^ in the unplanted reactor (Figure 3). The sulfadiazine removal efficiencies for the planted and unplanted reactors were 67.20% and 93.68%, respectively.The removal efficiency was higher in the unplanted reactor than in the planted reactor.

### 4.4. Isotherm Studies

Two models (the Langmuir and Freundlich models) were used to model the pharmaceutical adsorption of the Leca. These models are useful in full-scale applications.

1. Langmuir model

$$\begin{array}{l} original\;form\;Linearized\;form\\ \mathrm{1.}Langmuir\;model\\ \text{ q=}\frac{q_{m}.K_{L}.C}{1+K_{L}.C}\;\frac{C}{q}=\frac{1}{K_{Lq_{m}}}+\frac{1}{q_{m}}.C\\ \mathrm{2.}Freundlich\;model\\ q=K_{F.C^{1/n}\;}\log q=\log K_{F}+1/n\log C \end{array}$$

Where: Q: Pollutant quantity adsorbed per specific amount of adsorbent; C: Equilibrium concentration; q_m_: Pollutant quantity required to form a monolayer; K_L_: Langmuir equilibrium constant; (K_F_), and (1/n) are indicative isotherm parameters of sorption capacity and intensity, respectively (43,44).

**Figure 4:**
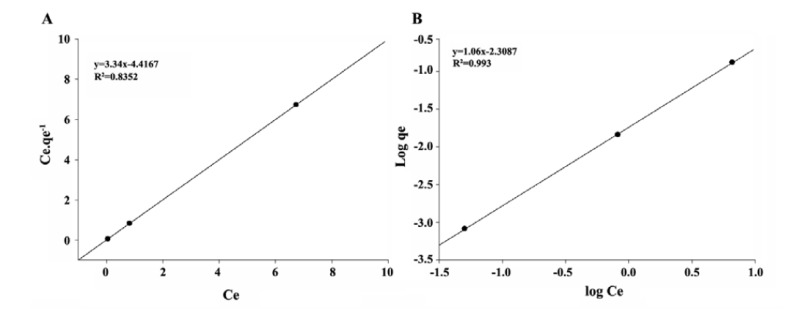
Carbamazepine adsorption A: Langmuir B: Freundlich. qe: Pollutant quantity adsorbed per specific amount of adsorbent; Ce: Equilibrium concentration

**Figure 5:**
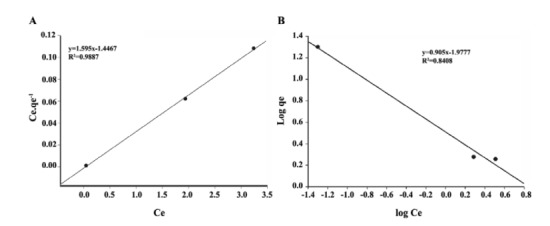
Ibuprofen adsorption A: Langmuir B: Freundlich. qe: Pollutant quantity adsorbed per specific amount of adsorbent; Ce: Equilibrium concentration

**Figure 6:**
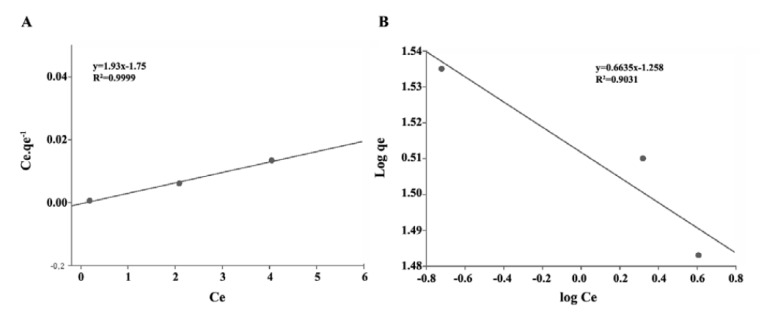
Sulfadiazine adsorption A: Langmuir B: Freundlich

Figures 4-6 show the adsorption of pharmaceuticals. The values of the Freundlich and Langmuir constants are presented in Table 1.

**Table 1:** The concentration of the carbamazepine, ibuprofen, and sulfadiazine in the Leca, body, and leaves of the *P. australis*

**The analysis section** **Pharmaceutical products**	** Influent concentration **	** Leca **	** P. australis body **	** P. australis leaf **
Carbamazepine μg.L^-1^ Ibuprofen μg.L^-1^ Sulfadiazine μg.L^-1^	58.43 33.07 33.39	46.58 <5 <5	43.75 <5 <5	154.55 <5 <5

## 5. Discussion

### 5.1. Carbamazepine Assays

Carbamazepine is not efficiently removed from wastewater in any treatment plant (45). Carbamazepine is one of the most durable pharmaceutical products in surface water (46). Plants have an active role in the removal of these compounds through direct uptake. It is widely accepted that organic compounds with 0.5 < log Kow < 3 can pass through cell membranes and enter plants’ transpiration streams, thereby easily being taken up by plants. In this study, high removal efficiencies were obtained for both the planted and unplanted reactors. It is thought that removal of carbamazepine (log Kow=2.45) from the used subsurface-flow system to have been caused by plant transpiration system and solid-phase biological sorption. As Table 2 is showing, the results obtained for carbamazepine, ibuprofen, and sulfadiazine concentration in the Leca, body, and the leaves of *P. australis* support this view. Carbamazepine was found to be adsorbed within Leca structure and in the body and leaves of *P. australis*, whereas ibuprofen and sulfadiazine were not. The carbamazepine concentrations in the leaves of the *P. australis* in Leca, and in the body of the *P. australis* were 154.55 μg.L^-1^, 46.58 μg.L^-1^, and 43.75 μg.L^-1^, respectively. Carbamazepine was adsorbed by the leaves of the *P. australis*, the Leca, and the body of the *P. australis* plants (in that order, from highest to the lowest adsorption). For carba mazepine, sorption of the dissolved organic contaminant in that organic matter and on the biofilm coating the Leca bed as well as *P. australis* could be a significant mechanism for their removal.

**Table 2:** Comparison between Langmuir and Freundlich isotherms models as obtained for the three types of the studied pharmaceuticals

** Constants **	** Langmuir Isotherm **			** Freundlich Isotherm **		
**Pharmaceutical products**	Q^o^	b	R^2^	N	KF	R^2^
Carbamazepine	0.805	0.29	0.8352	0.943	0.459	0.993
Ibuprofen	0.626	1.102	0.9887	1.104	0.457	0.8408
Sulfadiazine	0.518	1.102	0.8408	1.507	0.527	0.9031

### 5.2. Ibuprofen Assays

The removal efficiency of the ibuprofen in the treatment plants can be more than 90%, most likely due to the low hydrophobicity of the ibuprofen (logKow <3) (47). The biological transformation of ibuprofen is the main removal mechanism. However, the removal efficiencies in some wastewater treatment plants are reported to be in the order of 30% (48).

Consistent with the findings reported in the literature, higher removal efficiencies were achieved in this study in both the planted and unplanted reactors, as shown in Figure 2, indicating the concentrations of ibuprofen in the influent and effluent of the planted and unplanted reactors. The fact that ibuprofen did not accumulate in the Leca and *P. australis*, as shown in Table 2, suggests that other mechanisms such as biodegradation play a role in their removal. The ibuprofen concentrations in the leaves of the *P. australis* in Leca, and in the body of the *P. australis* were <5 μg.L^-1^ in all sample types.

### 5.3. Sulfadiazine Assays

It has been previously reported that sorption, abiotic transformation, and biotic transformation are the major processes that antibiotics undergo in the natural and engineered aquatic environments (49). Hydrolysis is an important degradation pathway for organic pollutants in aquatic environments (50). In this study, the removal of antibiotics was attributed to their biodegradation in water. It was shown that an unplanted system is more directly insulated and highly populated by microscopic algae, achieved significantly better removal than a planted system (51). It was found that sulfadiazine was removed at lower rates in planted systems (34). The sulfadiazine concentrations in the leaves of the *P. australis* in Leca, and in the body of the *P. australis* were <5 μg.L^-1^ in all sample types (Table 2). The higher degree of removal achieved in this study in the control reactor indicates that the presence of plants is not important in the removal of sulfadiazine.

### 5.4. Isotherm Studies

The adsorption of pharmaceuticals is better explained by Freundlich isotherm. In carbamazepine and sulfadiazine removal, the system shows compliance with the Freundlich isotherm model (R^2^ values of 0.993 and 0.9031, respectively). It is dependent on each of these drugs in the presence of a medicament for being adsorbed by the active sites on the surface that are distributed across a heterogeneous show that task. For the adsorption of sulfadiazine, the values for in see Table 1 were found to be greater than 1, which indicates that sulfadiazine is adsorbed better than the other studied pharmaceutical products. When the correlation coefficients (R^2^) of the model graphics are considered, a higher rate of carbamazepine was found to be in agreement with the Freund lich isotherm model. The Langmuir isotherm model did not adequately describe the removal of ibuprofen in the control tank. This degree of adsorption of ibuprofen indicates a monolayer adsorption (R^2^=9887).

## 6. Conclusions

According to the literature, carbamazepine, ibuprofen, and sulfadiazine are the most common and the most used pharmaceutical products found in domestic wastewater, surface, and underground water (52,53). In this study, the treatability of a laboratory-scale constructed wetland system used for removing pharmaceutical products was the subject of examination. The obtained results indicate that carbamazepine is adsorbed into the structure of Leca and the body and leaves of *P. australis*, whereas ibuprofen and sulfadiazine are not. While high removal efficiencies were obtained for both planted and unplanted reactors for carbamazepine and ibuprofen, in sulfadiazine assays, the removal efficiency was better for unplanted reactors compared to the planted reactors.

With respect to sulfadiazine and carbamazepine removal, the system exhibited compliance with the Freund lich isotherm model depending on each of these drugs in the presence of a medicament for the adsorption active sites on the surface that are distributed across a heterogeneous show that task. The Langmuir isotherm model did not adequately describe the removal of ibuprofen in the control tank. This degree of ibuprofen adsorption indicates a monolayer adsorption.

Based on the results obtained in this study, other concentrations and hydraulic retention times for pharmaceutical products should be examined in future studies, and more research is needed to be undertaken on the treatment of wastewater contaminated with the pharmaceutical products using constructed wetlands.

## Acknowledgments

This manuscript was edited by American Journal Experts (AJE). This study is a part of the PhD. Thesis of the first author, which was accepted on 19.12.2012 by the Graduate School of Natural and Applied Sciences of Uludag University.

## Funding/Support

This work was supported by grants from the Scientific Research Projects Council of Uludag University (Project number 2010/52), Bursa, Turkey.
